# Numerical Investigation on Tensile and Compressive Properties of 3D Four-Directional Braided Composites

**DOI:** 10.3390/ma18245592

**Published:** 2025-12-12

**Authors:** Longcan Chen, Feilong Dou, Jun Wang, Guangxi Li, Binchao Li, Jin Zhou, Yong Xue, Shenghao Zhang, Di Zhang

**Affiliations:** 1 National Key Laboratory of Aerospace Liquid Propulsion, Xi’an Aerospace Propulsion Institute, Xi’an 710100, China; volcanoacan@163.com (L.C.); junw83@163.com (J.W.); lbchmail@126.com (B.L.); 2 Ramjet Engine Research Laboratory of Stamping and Combined Power Division, Xi’an Aerospace Propulsion Institute, Xi’an 710100, China; doulf717@163.com; 3 School of Mechanical Engineering, Xi’an Jiaotong University, Xi’an 710049, China; jin.zhou@xjtu.edu.cn (J.Z.); zhangshenghao@stu.xjtu.edu.cn (S.Z.); 4 Technical Department, Xi’an Oude Rubber & Plastic Technology Co., Ltd., Xi’an 710200, China; xueyong@sxqc.com

**Keywords:** 3D braided composites, FE analysis, mechanical behavior, damage mechanisms, braiding angle

## Abstract

This study investigates the influence of braiding angles on the mechanical behavior and damage mechanisms of three-dimensional (3D) braided composites under uniaxial compressive and tensile loading. By integrating uniaxial compression and tension tests with finite element (FE) analysis, the relationships between mesoscale damage initiation, propagation, and the macroscopic mechanical properties were revealed. Results demonstrate that the 3D4d-20° model exhibits higher stiffness and compressive strength but lower compressive failure strain compared to the 3D4d-40° model, attributed to differences in fiber spatial arrangement and matrix cracking propagation. Conversely, the 3D4d-40° model shows enhanced tensile performance but greater matrix-dominated damage under tension. Moreover, as the braiding angle increases, the ratio of tensile strength to compressive strength in 3D braided composites decreases accordingly. Comparative analysis of damage evolution pathways reveals that smaller braiding angles (20°) initiate damage earlier under compression, while larger angles (40°) promote transverse fiber bundle failure and matrix degradation. This research not only elucidates the underlying microscale damage mechanisms of 3D braided composites under compression loading but also highlights the differences in damage patterns between compressive and tensile loading, providing theoretical foundations for structural design and performance optimization of such composite materials. Future work will focus on incorporating interfacial effects and manufacturing-induced defects to refine the model further.

## 1. Introduction

3D braided composites form an interwoven network structure through the 3D interlacing of fiber bundles, which effectively avoids the critical drawback of delamination commonly observed in conventional laminates. This structural characteristic endows the material with excellent mechanical strength and stiffness, superior energy absorption capacity, enhanced damage tolerance, and remarkable impact resistance [1,2,3,4]. Furthermore, it enables the single-step fabrication of complex configurations, making it particularly suitable for aerospace applications to meet the requirements of weight reduction and high load-bearing capacity [5,6,7,8,9,10]. Given the flexible and intricate spatial braided architecture of 3D braided composites, it is essential to investigate the influence of braiding structures on their mechanical properties and damage evolution mechanisms, thereby guiding the design and application of these materials.

Over the past three decades, extensive experimental studies have been conducted by both domestic and international researchers on 3D braided composites to investigate their mechanical properties and failure behavior. Lu et al. [11] investigated the compressive properties of 3D four-directional (3D4d) braided composites using macroscopic compression tests. Yan et al. [12] conducted macroscopic compression failure tests on thin plate specimens of 3D4d braided composites and analyzed the acoustic emission signals to study the compressive behavior and failure mechanisms of the material. Additionally, Li et al. [13] performed macroscopic and scanning electron microscopy (SEM) observations of the fracture surfaces of 3D five-directional braided composite specimens, examining the deformation and failure mechanisms from both macroscopic and mesoscopic perspectives. Guo et al. [14] conducted longitudinal tensile tests to study the mechanical behavior of 3D six-directional braided composites from a macroscopic perspective. Boris et al. [15] performed studies on the tensile properties of biaxial and triaxial 3D braided composites. Zhang et al. [16] carried out tensile and compressive tests on eight distinct braiding architectures of 3D braided composites. The results indicate that mechanical properties and failure behavior are closely related to the braiding architecture and braiding angles. The influence of the braiding architecture on the performance of these composites is reflected in variations in stress–strain responses, failure strains, and crack propagation paths.

With the advancement of FE analysis techniques, researchers worldwide have proposed various FE models. Ge et al. [17] developed an elasto-plastic damage coupling model to characterize the nonlinear mechanical behavior of 3D braided composites. This model can predict the initiation and propagation of fiber tow, as well as matrix degradation. Fang et al. [18] proposed a multi-scale coupling method integrating FE analysis with fast Fourier transform (FFT) to investigate the mechanical response of 3D braided composite beams with large braiding angles under bending loads. The results demonstrated that transverse damage in the fiber fabric emerged at the beam’s bottom as the applied load increased, accompanied by significant matrix degradation. These bottom-region damages were primarily attributed to matrix failure. To accurately predict the longitudinal tensile properties of 3D4d braided composites, Peng et al. [19] developed parametric mesoscopic solid models of face-cell and inner-cell architectures. The face-cell model incorporated yarn spatial trajectory offsets and cross-sectional extrusion deformation. Gao and Gereke et al. [20,21] systematically reviewed numerical simulation techniques for 3D braided composites.

Recent advancements in 3D braided composites have significantly expanded their engineering applications [22,23,24,25]. However, existing studies have predominantly focused on tensile performance characterization, with limited investigations dedicated to compressive behavior analysis. In particular, comprehensive research on compressive mechanical responses and damage evolution mechanisms of 3D braided composites through computational modeling remains limited. Consequently, an integrated approach combining FE numerical simulation with experimental validation to systematically investigate the influence of braiding angles and structural configurations on the mechanical properties and failure mechanisms of 3D braided composites represents a critical research direction with significant academic and engineering implications.

This study constructs unit-cell models of 3D4d-20° and 3D4d-40° 3D braided composites to systematically investigate the differential impacts of braiding angles on mechanical responses and damage evolution mechanisms under uniaxial compressive and tensile loading. By integrating experimentally validated simulation methods with uniaxial compressive/tensile test data, the distinct differences in key mechanical indicators—including stiffness, strength, and damage initiation strain—between the two models are quantitatively analyzed. A key finding is that the ratio of tensile strength to compressive strength decreases as the braiding angle increases. Through comparative analysis of damage evolution pathways under compressive and tensile loading, the governing role of braiding angles in determining composite failure modes is revealed. These findings, featuring the newly clarified variation law of tensile-compressive strength ratio alongside comprehensive mechanical property characterization, provide a more targeted and reliable theoretical foundation for optimizing the performance and engineering applications of 3D braided composites.

## 2. Materials and Tests

In the present study, 3D4d braided composites with theoretical internal braiding angles of 20° and 40° are investigated. The 3D braided fabrics were prepared using T700-3K carbon fiber yarns (Toray^®^, Tokyo, Japan) through the 4-step 1 × 1 procedure [26]. The preforms were then impregnated with a TDE86 resin matrix (Jingdong^®^, Tianjin, China) using the resin transfer molding (RTM) process.

**Figure 1:**
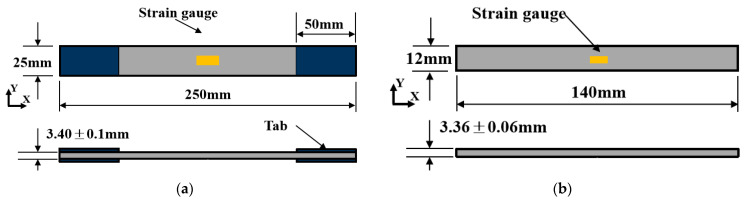
Sketch with the dimensions for (**a**) compressive and (**b**) tensile specimens.

Each test was conducted five times to improve the reliability of the final average data. The strain response of each specimen was recorded through strain gauges attached to the specimens, as shown in Figure 1. The longitudinal compressive tests were conducted using a combined loading compression (CLC) test fixture at room temperature. Following the ASTM D3039 [27] and ASTM D6641 [28] standards, an electronic CSS 44100 universal machine (CIMACH, Changchun, China) is used to conduct compressive and tensile tests with a displacement rate of 1 mm/min.

## 3. Methods

### 3.1. Material Properties

The fiber bundle contains fiber and matrix, which can be regarded as a transversely isotropic material. In this paper, the bridge model proposed by Huang [29] was used to predict the elastic properties of fiber bundles.

(1) $$\begin{array}{l} E_{X}=k_{f}E_{f1}+k_{m}E_{m}\\ \nu_{XY}=\nu_{XZ}=k_{f}\nu_{f12}+k_{m}\nu_{m}\\ \nu_{YZ}=(k_{f}\nu_{f23}E_{m}+k_{m}\nu_{m}E_{f2})/(k_{f}E_{m}+k_{m}E_{f2})\\ E_{Y}=E_{Z}=\frac{\left(k_{f}+k_{m}a_{11})(k_{f}+k_{m}a_{22}\right)}{\left(k_{f}+k_{m}a_{11})(k_{f}/E_{f2}+a_{22}k_{m}/E_{m})+k_{f}k_{m}(-\nu_{m}/E_{m}+\nu_{f12}/E_{f1}\right)}\\ G_{XY}=G_{XZ}=\frac{G_{f12}G_{m}(k_{f}+k_{m}a_{66})}{k_{f}G_{m}+k_{m}G_{f12}a_{65}}\\ G_{YZ}=\frac{G_{f23}G_{m}(k_{f}+k_{m}a_{22})}{k_{f}G_{m}+k_{m}G_{f23}a_{22}} \end{array}$$

where a_11_, a_22_, and a_66_ are the bridge connection numbers.

The fiber bundle is assumed to behave as a linear elastic material. Its strength parameters are obtained using the Chamis model [30], as shown in Equations (2)–(6).

The axial tensile strength is

(2) $$X_{t}=Xf_{t}(k_{f}+k_{m}\frac{E_{m}}{Ef_{1}})$$

The axial compressive strength is

(3) $$X_{c}=Xf_{c}(k_{f}+k_{m}\frac{E_{m}}{Ef_{1}})$$

The transverse tensile strength is

(4) $$Y_{t}=Z_{t}=(1-(\sqrt{k_{f}}-k_{f})(1-\frac{E_{m}}{Ef_{2}}))T_{m}$$

The transverse compressive strength is

(5) $$Y_{c}=Z_{c}=(1-(\sqrt{k_{f}}-k_{f})(1-\frac{E_{m}}{Ef_{2}}))C_{m}$$

The in-plane shear strength is

(6) $$S_{12}=S_{13}=(1-(\sqrt{k_{f}}-k_{f})(1-\frac{G_{m}}{Gf_{12}}))S_{m}$$

In Equations (2)–(6), *X*_t_ and *X*_c_ denote the tensile and compressive strengths of yarn in its longitudinal direction, respectively, while *Y*_t_, *Z*_t_, *Y*_c_, and *Z*_c_ denote the corresponding tensile and compressive strengths perpendicular to the yarn direction. The shear strengths in the 1–2 and 1–3 planes are presented by *S*_12_ and *S*_23_, respectively. The Chamis model cannot be used to predict the shear strength in the 2–3 plane. Therefore, the empirical method proposed by Christensen [31] is employed to calculate *S*_23_, as shown below:

(7) $$S_{23}=\sqrt{Y_{t}Y_{c}\frac{1+Y_{t}/Y_{c}}{3+5Y_{t}/Y_{c}}}$$

The outer matrix of the fiber bundle is regarded as an isotropic material. The material properties in different directions are the same. Table 1 and Table 2 are material parameters of T700 carbon fiber and TDE86 epoxy resin.

### 3.2. Progressive Damage Model

In the past few decades, the Hashin criterion has been widely used and has maintained high accuracy in the damage prediction of composite materials [32,33]. Therefore, the Hashin criterion [34] is used to predict the longitudinal tensile damage of the fiber bundle; these criteria are given as:

(8) $$F_{\mathrm{xt}}^{2}={\left(\frac{\sigma_{11}}{X_{t}}\right)}^{2}+\frac{{\sigma_{12}}^{2}}{{S_{12}}^{2}}+\frac{{\sigma_{13}}^{2}}{{S_{13}}^{2}}\geq 1,\;(\sigma_{11}\geq 0)$$

where $\sigma_{11}$, $\sigma_{12}$, and $\sigma_{13}$ correspond to the normal stress in the X direction and the shear stresses in the XY and XZ planes, respectively.

For 3D braided composites, the fiber bundle has a certain inclination in space, and the effect of shear stress cannot be ignored. Therefore, for the axial compressive failure of the fiber bundle, the same formulation as that for axial tensile failure is adopted:

(9) $$F_{\mathrm{xc}}^{2}={\left(\frac{\sigma_{11}}{X_{c}}\right)}^{2}+\frac{{\sigma_{12}}^{2}}{{S_{12}}^{2}}+\frac{{\sigma_{13}}^{2}}{{S_{13}}^{2}}\geq 1,\;(\sigma_{11}<0)$$

For damage of the fiber bundle in the transverse (Y-direction) and normal (Z-direction) directions, based on the Hou failure criteria [35]:

(10) $$F_{\mathrm{yt}}^{2}={\left(\frac{\sigma_{22}}{Y_{t}}\right)}^{2}+\frac{{\sigma_{12}}^{2}}{{S_{12}}^{2}}+\frac{{\sigma_{23}}^{2}}{{S_{23}}^{2}}\geq 1,\;(\sigma_{22}\geq 0)$$

(11) $$F_{\mathrm{yc}}^{2}={\left(\frac{\sigma_{22}}{Y_{c}}\right)}^{2}+\frac{{\sigma_{12}}^{2}}{{S_{12}}^{2}}+\frac{{\sigma_{23}}^{2}}{{S_{23}}^{2}}\geq 1,\;(\sigma_{22}<0)$$

(12) $$F_{\mathrm{zt}}^{2}={\left(\frac{\sigma_{33}}{Z_{t}}\right)}^{2}+\frac{{\sigma_{13}}^{2}}{{S_{13}}^{2}}+\frac{{\sigma_{23}}^{2}}{{S_{23}}^{2}}\geq 1,\;(\sigma_{33}\geq 0)$$

(13) $$F_{\mathrm{zc}}^{2}={\left(\frac{\sigma_{33}}{Z_{c}}\right)}^{2}+\frac{{\sigma_{13}}^{2}}{{S_{13}}^{2}}+\frac{{\sigma_{23}}^{2}}{{S_{23}}^{2}}\geq 1,\;(\sigma_{33}<0)$$

where $\sigma_{22}$, $\sigma_{33}$, and $\sigma_{23}$ are the normal stress in the Y-and Z-directions, and the shear stress in the yz plane, respectively.

The matrix will also undergo an elastic deformation stage, an initial failure stage, and a damage degradation stage in the actual damage process. The matrix damage is modeled using the damage criterion proposed by Christensen [36], which has been demonstrated to effectively predict the damage in isotropic materials under various stress states.

(14) $$\begin{array}{l} {F_{m}}^{2}=(\frac{1}{T_{m}}-\frac{1}{C_{m}})(\sigma_{11}+\sigma_{22}+\sigma_{33})+\frac{1}{T_{m}C_{m}}\{\frac{1}{2}[{\left(\sigma_{11}-\sigma_{22}\right)}^{2}\\ +(\sigma_{22}-\sigma_{33}{)}^{2}+{\left(\sigma_{33}-\sigma_{11}\right)}^{2}+3({\sigma_{12}}^{2}+{\sigma_{13}}^{2}+{\sigma_{23}}^{2})\}\geq 1 \end{array}$$

### 3.3. Damage Evolution Model

When the corresponding damage criterion is satisfied, the stiffness of the material will decrease gradually in the course of further loading. The process of stiffness reduction is described by a damage variable, which changes from 0 (indicating a completely undamaged state) to 1 (representing a completely damaged state), reflecting the degree of development of the material damage.

The introduction of the characteristic length can effectively reduce the mesh dependence in the FE analysis. Specific values of $G_{i}$ are shown in Table 3 and Table 4. The tensile and compressive fracture energies of fiber bundles Y and Z are equal.

To mitigate the challenges of convergence in an implicit analysis, the Duvaut–Lions regularization [38] is adopted here. The stiffness matrix of the material is reduced by the Murakami–Ohno model [39].

## 4. Mesoscopic FE Model

The mesoscale FE model accurately captures the true textile architecture of composite fabrics by geometrically distinguishing between fiber bundles and pure matrix regions. By assigning distinct material properties and failure criteria to the fiber bundles and matrix, this approach is well-suited for analyzing stress distribution and damage evolution in textile composites. The 3D braided composite mesoscale model established in this study is based on the actual geometric configuration of the representative volume element (RVE), utilizing fully hexahedral meshing to minimize stress concentrations, element distortions, and to achieve high computational efficiency. Furthermore, the implementation of a 3D failure criterion capable of distinguishing different damage modes enables the simulation of realistic localized deformation, stress–strain distribution, and the initiation/propagation of various damage mechanisms.

### 4.1. Geometric Structure of Unit-Cell

**Figure 2:**
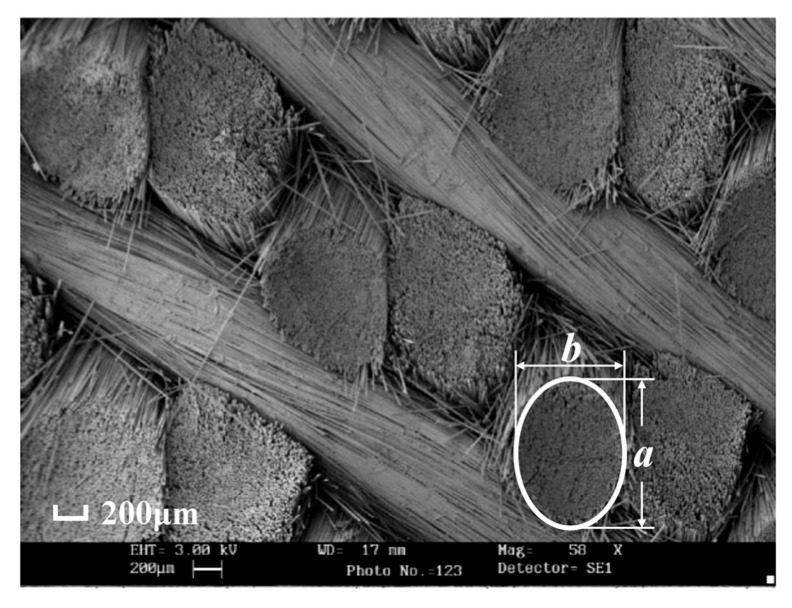
Preform cross-section of 3D4d braided composite [40].

In the braided preform, the braiding yarn is composed of fiber bundles. As shown in Figure 2, the cross-section of the 3D4d braided preform reveals that the fiber bundles exhibit independent spatial arrangements, with their cross-sections primarily elliptical in shape.

**Figure 3:**
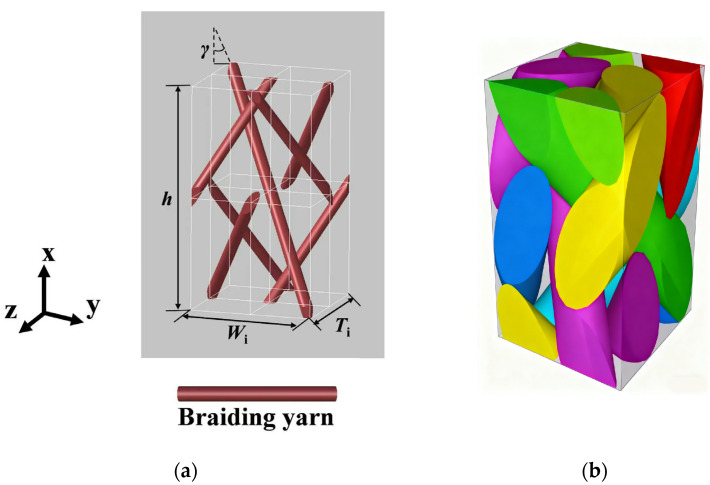
Interior unit-cell and solid model of 3D4d braided composites: (**a**) Interior unit-cell model, (**b**) FE model.

The 3D braided composite element structure model proposed by Wu [41] is adopted in this paper, as shown in Figure 3a. Considering the extrusion deformation of the yarns, several assumptions are applied to model the geometrical configurations. Based on the geometric relationships illustrated in the figure, the geometric dimensions of the internal unit cells and the correlations among related parameters can be analyzed to determine:

(15) $$T_{i}=W_{i}=4\sqrt{2}b$$

(16) H=8b/tanγ

(17) $$a=b\sqrt{3}\cos\gamma$$

The spatial positions of yarns are calculated using MATLAB R2021b based on the mesoscale geometric model of the unit cell. Subsequently, the mesoscale unit cell CAE model for Abaqus can be automatically generated through Texgen v3.13.1 software, as shown in Figure 3b.

### 4.2. Boundary Conditions

Appropriate boundary conditions are essential for enhancing the accuracy of FE analysis. This study employs the periodic boundary conditions (PBCs) proposed by Li [42,43]. PBCs ensure that corresponding nodes on opposite faces of each RVE align, preserving the periodicity and continuity of deformation. To maintain the structural periodicity of the deformed RVE, Python scripts were developed in ABAQUS 2017 to enforce PBCs on the RVE model, where corresponding mesh nodes are generated on parallel faces to apply the boundary conditions.

### 4.3. Mesh Discretization

The mesh size, defined in Texgen, is approximately 0.1 mm, ensuring converged computational results. The unit cell model comprises 20 × 20 × 30 elements, all of which are eight-node solid elements with reduced integration (C3D8R). Constraints are applied using periodic boundary conditions, and a prescribed loading rate is applied to simulate the uniform compressive loading conditions of the experimental setup.

## 5. Results

Based on the 3D braided composites mesoscopic FE model, the compressive and tensile simulation analyses are carried out, and the progressive damage behavior and failure mechanism are discussed in combination with the experimental results.

### 5.1. Mechanical Behavior

**Figure 4:**
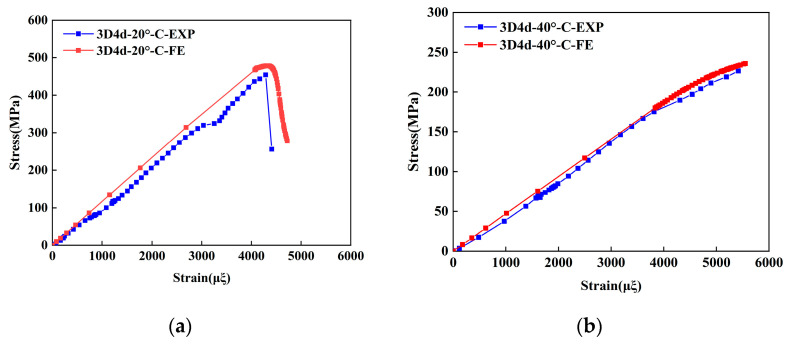
Comparison of the predicted results with the experimental data under longitudinal compressive loading: (**a**) 3D4d-20°; (**b**) 3D4d-40°.

**Figure 5:**
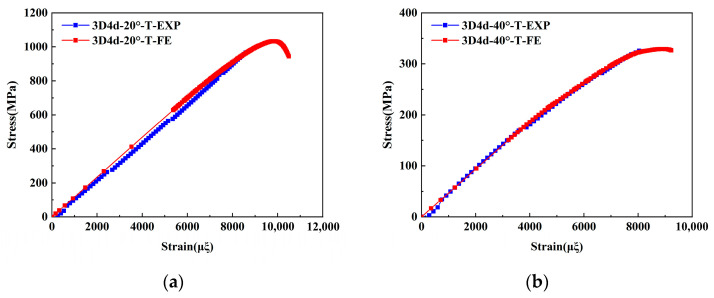
Comparison of the predicted results with the experimental data under longitudinal tensile loading: (**a**) 3D4d-20°; (**b**) 3D4d-40°.

To validate the proposed FE model, the longitudinal compressive and tensile responses of 3D4d braided composites with different braiding angles were compared with experimental data, as shown in Figure 4 and Figure 5, and Table 5.

The results indicate that the predicted longitudinal compressive stress–strain curves initially exhibit a linear increase, followed by a rapid decline in peak stress as strain increases. The model accurately captures the brittle fracture behavior of the three types of composites, consistent with experimental observations. Notably, the maximum deviation between the FE model predictions and experimental data remains below 8%. Furthermore, the compressive modulus and strength decrease with increasing braiding angle. This is attributed to the increased irregularity of fiber spatial arrangement at larger braiding angles, which reduces the effective stiffness of the material and results in more pronounced nonlinear behavior in the stress–strain curves. These phenomena align with the experimental conclusions from Beihang University [11]. Regarding compressive failure strain, the failure strain increases with the braiding angle. This is primarily due to smaller braiding angles promoting matrix cracking and fiber-matrix debonding under compressive loading, which causes fiber bundle instability and premature loss of load-bearing capacity at lower strain levels. It is worth noting that the unit cell model does not account for interface failure between the tows and the matrix, explaining consistently higher simulated strength and stiffness values compared to experimental data. Under tensile loading, the simulation results demonstrate a good agreement with the experimental data. For both braiding angles, the simulated failure strain and stress of the 3D4d braided composites are consistently higher than their experimental values.

Experimental and numerical studies on 3D braided composites have consistently demonstrated that the tensile modulus and strength decrease monotonically with increasing braiding angle. This is attributed to the reduced effective fiber alignment along the loading axis at larger angles, which decreases the structural efficiency of the reinforcement. Conversely, smaller braiding angles improve fiber continuity and reduce stress concentrations, leading to higher tensile failure strain. However, larger braiding angles increase susceptibility to matrix cracking and interfacial debonding under tensile loading, an effect exacerbated by the Poisson effect, resulting in lower failure strain. This behavior is consistent with micro-mechanical analyses reported in the prior study [44]. Notably, the numerical predictions obtained from FE analysis closely match the experimental data, with the maximum deviation between simulated and measured results remaining below 8%. This low error margin validates the accuracy of the computational models in capturing the anisotropic mechanical behavior of 3D braided composites.

**Figure 6:**
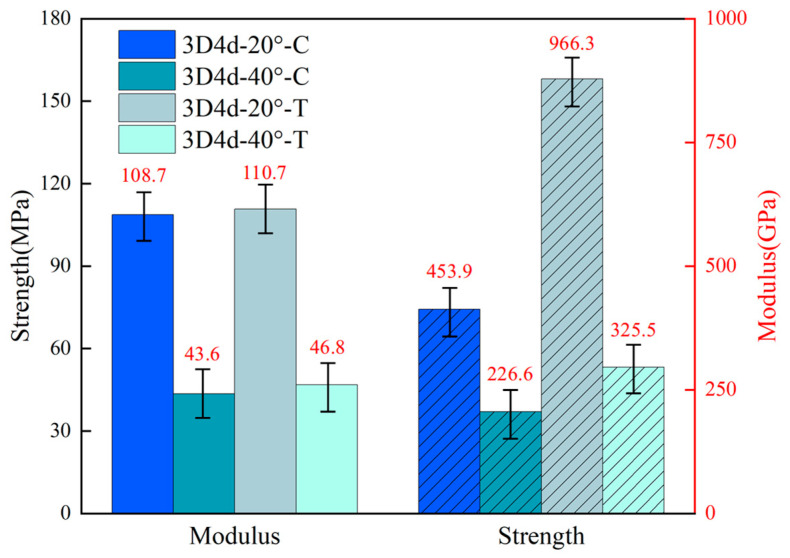
Comparison of experimentally tested modulus and strength of 3D braided composites with different braiding angles.

The mean and variance of longitudinal tensile and compressive properties of 3D braided composites with two different braiding angles are compared in Figure 6. An examination of the figure shows that the ratio of tensile strength to compressive strength decreases while the braiding angle increases. Specifically, the tensile strength of 3D4d-20°-T was 2.1 times as much as its compressive strength. However, the tensile strength of 3D4d-40°-T was 1.4 times as much as its compressive strength. A similar phenomenon is reported in the prior study [45]. The explanation for this phenomenon starts with the fiber orientation effect: at small braiding angles (20°), fibers are close to the loading direction, enabling high efficiency under tensile loading; while for compressive loading, the nearly parallel fibers tend to undergo fiber microbuckling, resulting in relatively low compressive strength. At large braiding angles (40°), fibers deviate from the load direction, reducing tensile efficiency; however, under compressive loading, the inclined fiber structure provides better lateral support to enhance buckling resistance, and meanwhile, compressive failure is more controlled by matrix shear, with the difference between the tensile and compressive strengths of the matrix usually smaller than that dominated by fibers. Another explanation lies in the transition of failure modes: at small braiding angles, tensile failure is dominated by fiber breakage, while compressive failure is dominated by fiber buckling, showing significant differences between the two mechanisms; at large braiding angles, both tensile and compressive failures are predominantly controlled by matrix shear and interface failure, making the two mechanisms more similar. The moduli of specimens with two braiding angles are approximately equivalent under tensile and compressive loads.

### 5.2. Failure Modes

**Figure 7:**
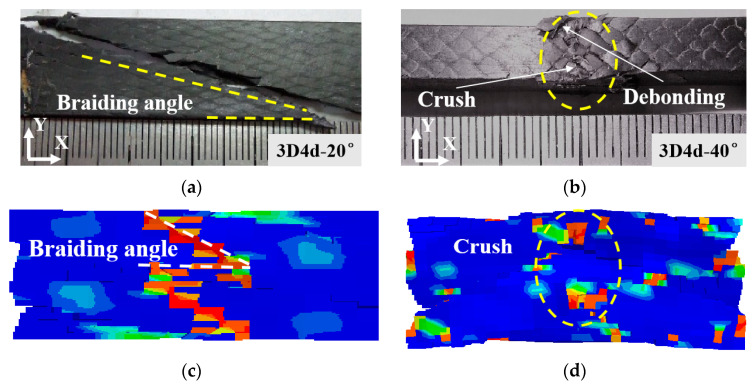
Compressive failure of 3D4d braided composites with two different braiding angles: (**a**) 3D4d-20° (Exp); (**b**) 3D4d-40° (Exp); (**c**) 3D4d-20° (FE); (**d**) 3D4d-40° (FE).

The experimental results demonstrate that the 3D4d-20° specimen exhibits significantly lower transverse load-bearing capacity compared to the 3D4d-40° specimen. This disparity directly dictates the final failure mode of the material, aligning with the failure mechanisms reported in previous studies [11,13]. The consistent failure pattern reveals the intrinsic stress distribution characteristics within the material under loading. Figure 7 depicts the external damage morphologies of 3D braided composites under compressive loading. The compressive failure of the 3D4d-20° specimen occurs predominantly through shear-dominated failure along a direction approximately aligned with the internal braiding angle, whereas the 3D4d-40° specimen exhibits failure dominated by central crushing and matrix–fiber debonding. This damage mechanism highlights the complex stress distribution and multi-stage damage evolution processes observed in the material under external loading conditions. In contrast, tensile failure is primarily characterized by fiber fracture perpendicular to the loading direction. The 3D4d-20° specimen undergoes shear-dominated failure along the braiding direction, while also exhibiting fiber pull-out phenomena. These findings highlight that a larger braiding angle facilitates matrix–fiber interface debonding under tensile loading, ultimately resulting in fiber fracture perpendicular to the loading direction.

**Figure 8:**
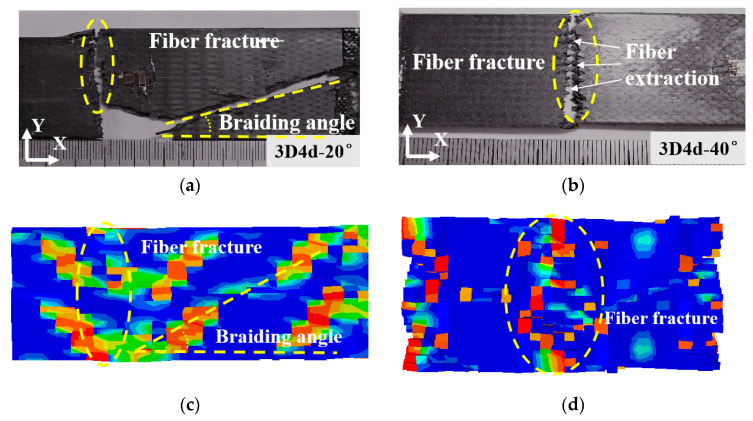
Tensile failure of 3D4d braided composites with two different braiding angles: (**a**) 3D4d-20° (Exp); (**b**) 3D4d-40° (Exp); (**c**) 3D4d-20° (FE); (**d**) 3D4d-40° (FE).

The predicted damage patterns, depicted in Figure 7c,d, and Figure 8c,d, exhibit excellent agreement with experimental observations. The contour color reflects failure state of units in 3D braided composites, where undamaged units appear blue and completely failed units appear red. These findings confirm the accuracy of the proposed FE model in predicting the mechanical behavior of 3D4d braided composites under longitudinal loading.

### 5.3. Prediction of Damage Mechanism

**Figure 9:**
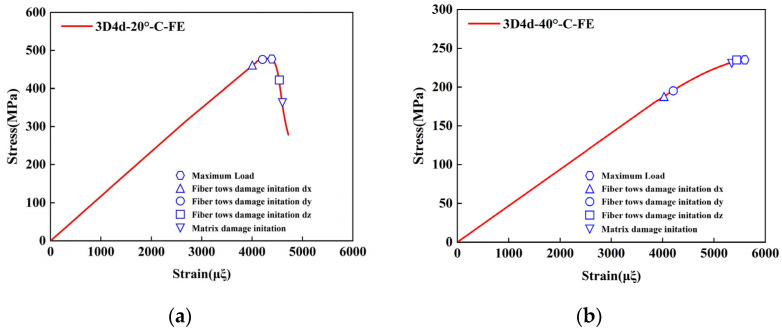
Damage evolution process of the unit-cell model with two different braiding angles under longitudinal compressive loading: (**a**) 3D4d-20°; (**b**) 3D4d-40°.

Figure 9a presents the stress–strain curve of the unit cell model for 3D4d braided composites with a 20° braiding angle under longitudinal compressive loading. As the strain increases, the fiber bundles (primary load-bearing components) initiate damage at 0.397% strain. The damage progresses gradually, with fiber bundle dy failing at 0.42% strain and the material achieving its ultimate strength (maximum stress) at 0.432% strain. Subsequently, brittle fracture occurs, leading to a rapid reduction in load-bearing capacity. This mechanical response is consistent with findings from previous studies [13,46]. At 0.454% strain, the fiber bundle dz sustains damage, while matrix cracking is observed at 0.46% strain. The variables dx, dy, and dz denote the damage components along the X, Y, and Z directions, respectively. The damage evolution process for the unit cell model with a 40° braiding angle is illustrated in Figure 9b.

**Figure 10:**
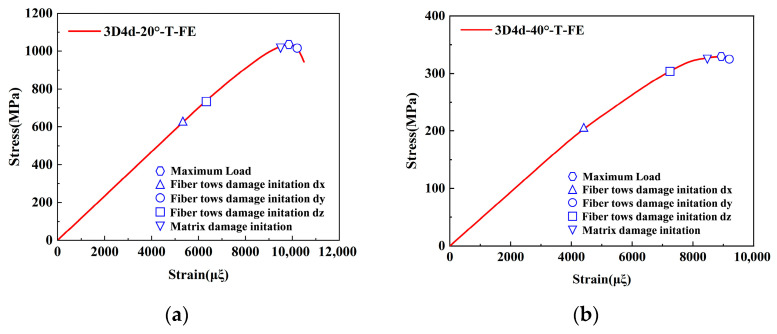
Damage evolution process of the unit-cell model with two different braiding angles under longitudinal tensile loading: (**a**) 3D4d-20°; (**b**) 3D4d-40°.

Figure 10 illustrates the damage evolution process of a 3D4d braided composite under longitudinal tensile loading. In 3D braided composites, fiber tows serve as the primary load-bearing components, and their damage extent and evolution mechanisms are critical to the analysis. This and subsequent analyses will focus on the evolution of fiber tow dx damage under different loading levels, while also presenting the final damage states of fiber tows dy and dz, as well as the matrix damage.

**Figure 11:**
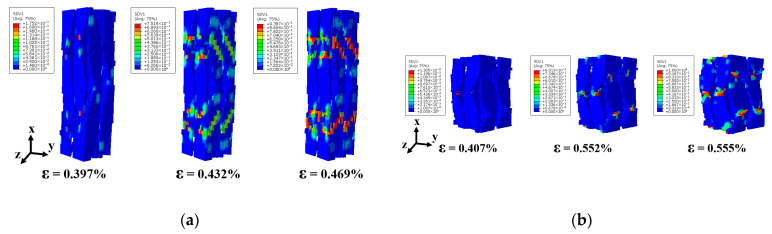
Comparison of damage evolution in the X direction of fiber bundles with two different braiding angles under longitudinal compressive loading: (**a**) 3D4d-20°; (**b**) 3D4d-40°.

Figure 11 compares the X-directional damage evolution of fiber bundles under compressive loading in two single-cell models with different internal braiding angles. Figure 11a illustrates the dx damage states of fiber bundles in the single-cell model under longitudinal compression at strain levels of 0.397%, 0.432%, and 0.469%. The results indicate that the initial dx damage occurs in regions of mutual fiber squeezing, a phenomenon consistently observed in both single-cell model simulations. However, the subsequent damage evolution in the fiber bundle differs between the models. For the 3D4d-20° model, the damage in the fiber bundles primarily originates from the initial damage location and propagates along the fiber axis. When the strain reaches 0.469%, the filaments exhibit severe damage along the entire braiding direction of the fiber bundle, indicating filament fracture. For the 3D4d-40° model, the damage in the fiber bundles propagates vertically along the fiber. As shown in Figure 11a,b, compared to the model with larger braiding angles, the smaller braiding angle model exhibits earlier damage onset and a significantly greater number of units undergoing axial compressive failure. This is attributed to higher stress levels and greater load-bearing capacity within the fiber bundles, resulting in faster overall material degradation and earlier damage initiation.

**Figure 12:**
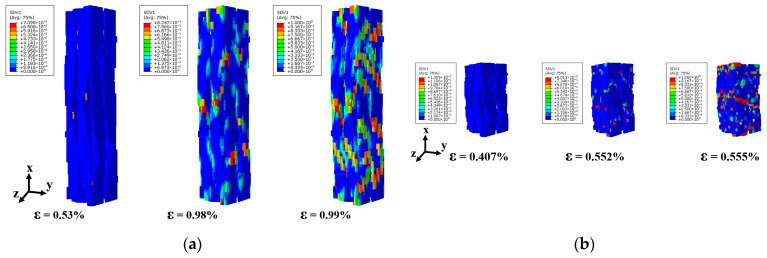
Comparison of damage evolution in the X direction of fiber bundles with two different braiding angles under longitudinal tensile loading: (**a**) 3D4d-20°; (**b**) 3D4d-40°.

As shown in Figure 12, the X-directional damage evolution of fiber bundles in two unit-cell models with different internal braiding angles under tensile loading is compared. The results reveal that, similar to the compressive loading case, the initial dx damage in the fiber bundles predominantly occurs at positions where fibers are subjected to mutual compression. For the 3D4d-20° model, damage propagates primarily along the X-axis, whereas in the 3D4d-40° model, damage evolution follows a direction perpendicular to the fiber bundles.

**Figure 13:**
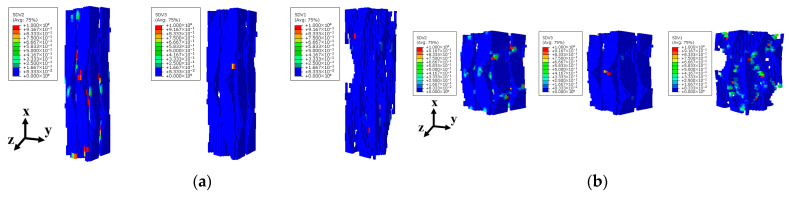
Comparison of damage for matrix and fiber bundles in the Y and Z directions with two different braiding angles under longitudinal comprehensive loading: (**a**) 3D4d-20°; (**b**) 3D4d-40°.

**Figure 14:**
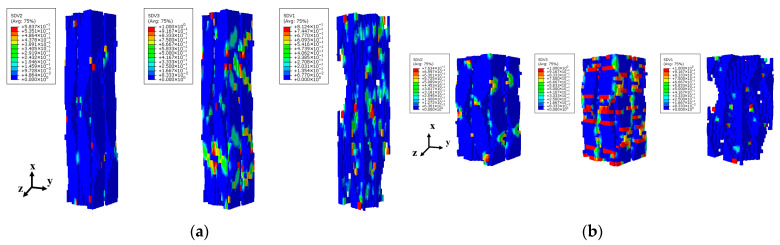
Comparison of damage for matrix and fiber bundles in the Y and Z directions with two different braiding angles under longitudinal tensile loading: (**a**) 3D4d-20°; (**b**) 3D4d-40°.

Figure 13 presents the transverse damage (dy, dz) in fiber bundles and matrix of single-cell models with different internal braiding angles under strain levels of 0.469%, 0.555%, 0.497%, and 0.554%. The results reveal that smaller braiding angles correlate with more pronounced transverse fiber bundle damage, indicating reduced contribution of fiber bundles to transverse mechanical performance—consistent with findings from previous studies by the research team at Beihang University [11]. These observations highlight significant differences in transverse damage characteristics among 3D4d braided fiber bundles under different braiding angles. Notably, the 3D4d-40° model exhibits greater matrix damage compared to the 3D4d-20° model, indicating a more pronounced matrix-dominated load-bearing behavior in the 40° configuration. Figure 14 illustrates the dy and dz of the fiber bundle and matrix of single-cell models with varying internal braiding angles under longitudinal tensile loading. Both models exhibited relatively high damage levels in the Z direction. However, significant differences were observed in the damage propagation directions: in the 3D4d-20° model, damage propagated along the warp angle, whereas in the 3D4d-40° model, damage propagated perpendicular to the fiber bundle axis.

Although the integration of experimental investigations with micro-scale modeling has provided valuable insights into the mechanical behavior of 3D braided composites, certain limitations should be acknowledged. The geometric representation of braided structures in the models remains idealized, neglecting manufacturing-induced variations and defects. Furthermore, interfacial interactions between fiber bundles and the matrix are not considered in the current framework. Future research should prioritize the development of more sophisticated geometric representations that incorporate manufacturing-induced imperfections, combined with the explicit consideration of interfacial effects, to improve simulation accuracy. Additionally, in situ compression testing will be carried out in future studies to experimentally observe and document the damage evolution process of 3D braided composites, thereby strengthening the credibility of computational simulations through experimental validation.

## 6. Conclusions

In this study, the mechanical behavior and damage mechanisms of 3D4d braided composites with internal braiding angles of 20° and 40° are investigated through experimental tests and micro-scale FE models. Longitudinal compressive and tensile tests are conducted, and the FE model is validated through experimental data to analyze stress–strain responses, damage evolution, and failure mechanisms. The following conclusions are drawn:

(1) The proposed micro-scale FE model accurately predicts the longitudinal compressive and tensile responses of 3D4d braided composites, with a maximum deviation of less than 8% from experimental data. This validates the model’s ability to capture the anisotropic mechanical behavior and brittle fracture characteristics of the composites under various loading conditions, providing a reliable computational tool for optimizing design parameters and reducing experimental costs.
(2) The compressive modulus and strength of 3D4d braided composites decrease with increasing internal braiding angle, while the compressive failure strain increases. This is attributed to the irregular spatial arrangement of fibers at larger braiding angles, which reduces the effective stiffness but enhances the strain capacity. Conversely, under tensile loading, the tensile modulus and strength decrease with increasing braiding angle, attributed to reduced fiber alignment along the loading axis. Smaller braiding angles enhance fiber continuity and increase the tensile failure strain, while larger angles promote matrix cracking and interfacial debonding. These findings highlight the trade-off between stiffness and ductility governed by braiding geometry, offering valuable guidance for optimizing material performance in engineering applications.
(3) The tensile-to-compressive strength ratio of 3D four-directional braided composites exhibits a significant decrease with increasing braiding angle. This reduction stems from a fundamental transition in failure mechanisms: at low braiding angles, failure is dominated by fiber fracture in tension and fiber micro-buckling in compression, representing distinct mechanisms. In contrast, at high braiding angles, both tensile and compressive failures become governed by matrix shear and interfacial debonding, resulting in convergent failure modes.
(4) Distinct damage mechanisms are observed in composites with different braiding angles: 3D4d-20° composites exhibit fiber bundle axial fracture and matrix cracking under compression, whereas 3D4d-40° composites show matrix-dominated damage and central crushing. Under tensile loading, 3D4d-20° composites experience fiber pull-out and shear failure, whereas 3D4d-40° composites exhibit perpendicular fiber fracture. These differences underscore the critical role of braiding angle in governing damage propagation paths and failure mechanisms, which is essential for predicting service-life performance and enhancing structural reliability.

Although this finite element model can effectively characterize the stress–strain response and damage evolution behavior of 3D braided composites, it has not yet considered the interface effect between fibers and the matrix. Future research should incorporate an interface model to further improve the accuracy of the model. In addition, it is advisable to adopt more refined geometric modeling approaches to account for manufacturing defects and material variability, thereby achieving more precise numerical simulations. We could leverage computer vision by using high-resolution image analysis [47,48] to provide pixel-level validation of the FE-predicted damage evolution and failure modes.

## Figures and Tables

**Table 1:** Mechanical parameters of T700 carbon fiber [6].

	T700
$\mathrm{Elastic}\;\mathrm{modulus}\;Ef_{1}$/GPa	230
$\mathrm{Elastic}\;\mathrm{modulus}\;Ef_{2}(Ef_{3})$/GPa	18.2
$\mathrm{Poisson}’s\;\mathrm{ratio}\;\nu f_{12}$	0.27
$\mathrm{Poisson}’s\;\mathrm{ratio}\;\nu f_{12}(\nu f_{23})$	0.30
$\mathrm{Shear}\;\mathrm{modulus}\;Gf_{12}(Gf_{13})$/GPa	36.6
$\mathrm{Shear}\;\mathrm{modulus}\;Gf_{23}$/GPa	7.0
$\mathrm{Tensile}\;\mathrm{strength}\;Xf_{1t}$/GPa	4.9
$\mathrm{Compression}\;\mathrm{strength}\;Xf_{1c}$/GPa	1.4

**Table 3:** Fracture energy densities of fiber tows [37].

G_Xt_ (N/mm)	G_Xc_ (N/mm)	G_Yt_ (N/mm)	G_Yc_ (N/mm)
12.78	6.26	0.13	1.15

**Table 4:** Fracture energy densities of matrix [37].

G_mt_ (N/mm)	G_mc_ (N/mm)
1.0	1.0

**Table 2:** Mechanical parameters of TDE86 epoxy resin [6].

	TDE86
$\mathrm{Elastic}\;\mathrm{modulus}\;E_{m}$/GPa	3.45
$\mathrm{Poisson}’s\;\mathrm{ratio}\;\nu_{m}$	0.35
$\mathrm{Tensile}\;\mathrm{strength}\;T_{m}$/GPa	0.08
$\mathrm{Compression}\;\mathrm{strength}\;C_{m}$/GPa	0.180
$\mathrm{Shear}\;\mathrm{strength}\;S_{m}$/GPa	0.2

**Table 5:** Comparison of modulus and strength between the predicted results and the experimental data.

	Modulus (GPa)			Strength (MPa)		
Test	FE	EXP	Relative Error	FE	EXP	Relative Error
3D4d-20°-C	114.8	108.7	5.6%	478.4	453.9	5.4%
3D4d-40°-C	46.9	43.6	7.6%	236	226.6	4.1%
3D4d-20°-T	117.2	110.7	6.0%	1032.5	966.3	6.4%
3D4d-40°-T	49.5	46.8	5.4%	326.2	325.5	0.3%

C or T corresponds to compression or tension.

## Data Availability

The original contributions presented in this study are included in the article. Further inquiries can be directed to the corresponding authors.
